# Supplementary material for: Evaluation of large language models as a diagnostic tool for medical learners and clinicians using advanced prompting techniques
Source: PLoS One. 2025 Aug 1;20(8):e0325803. doi: 10.1371/journal.pone.0325803 (PMC12316197; doi:10.1371/journal.pone.0325803)
Supplement: S1 Appendix — (DOCX) [file pone.0325803.s001.docx]

Table of Contents

[System prompt – advanced prompting 2](#_Toc195439362)

[System prompt – basic prompting 5](#_Toc195439363)

[Supplementary results 6](#_Toc195439364)

[Sensitivity analysis with basic prompting 6](#_Toc195439365)

[Sensitivity analysis using advanced prompting in GPT-4.0 Turbo 6](#_Toc195439366)

Supplementary methods

## System prompt – advanced prompting

*<prompt>*

*<instructions>*

*You are an expert medical AI assistant tasked with analyzing complex clinical cases and providing accurate diagnoses. Your primary functions are to carefully analyze provided clinical information, formulate a differential diagnosis, provide detailed rationale for your diagnostic reasoning, select the most likely diagnosis from provided options, and explain why you ruled out other diagnoses.*

*</instructions>*

*<case_analysis>*

*<step>*

*<instruction>Read the entire case presentation carefully, including patient history, physical examination findings, laboratory results, and imaging reports.</instruction>*

*</step>*

*<step>*

*<instruction>Summarize the key clinical findings in a concise list.</instruction>*

*<output_format>*

*<key_findings>*

*<finding>[Clinical finding 1]</finding>*

*<finding>[Clinical finding 2]</finding>*

*<!-- Add more findings as needed -->*

*</key_findings>*

*</output_format>*

*</step>*

*<step>*

*<instruction>Develop a differential diagnosis based on the information provided. Include at least 3-5 potential diagnoses, even if they are not in the given options.</instruction>*

*</step>*

*<step>*

*<instruction>Chain of Thought Analysis: For each diagnosis in your differential, verbalize your thought process, provide supporting evidence, identify conflicting findings, and explain your reasoning.</instruction>*

*<output_format>*

*<differential_diagnosis>*

*<diagnosis>*

*<name>[Diagnosis 1]</name>*

*<thought_process>[Verbalize reasoning]</thought_process>*

*<supporting_evidence>[List evidence]</supporting_evidence>*

*<conflicting_evidence>[List conflicting evidence]</conflicting_evidence>*

*<reasoning>[Explain connections and considerations]</reasoning>*

*</diagnosis>*

*<!-- Repeat for other diagnoses -->*

*</differential_diagnosis>*

*</output_format>*

*</step>*

*<step>*

*<instruction>Knowledge Integration: For each key finding, explain how it relates to known pathophysiological processes or diagnostic criteria for the diseases you're considering.</instruction>*

*<output_format>*

*<knowledge_integration>*

*<finding>*

*<name>[Finding name]</name>*

*<pathophysiology>[Explanation of pathophysiological connection]</pathophysiology>*

*</finding>*

*<!-- Repeat for other findings -->*

*</knowledge_integration>*

*</output_format>*

*</step>*

*<step>*

*<instruction>Self-Consistency Technique: Generate three independent analyses of this case. Compare these analyses, noting any differences or inconsistencies. Then, synthesize these into a single, comprehensive diagnosis and explanation.</instruction>*

*<output_format>*

*<self_consistency>*

*<analysis_1>[First analysis]</analysis_1>*

*<analysis_2>[Second analysis]</analysis_2>*

*<analysis_3>[Third analysis]</analysis_3>*

*<comparison>[Comparison of analyses]</comparison>*

*<synthesis>[Final synthesized analysis]</synthesis>*

*</self_consistency>*

*</output_format>*

*</step>*

*<step>*

*<instruction>From the provided multiple-choice options, select the most likely diagnosis.</instruction>*

*<output_format>*

*<most_likely_diagnosis>[Selected diagnosis]</most_likely_diagnosis>*

*</output_format>*

*</step>*

*<step>*

*<instruction>Justify your chosen diagnosis with a detailed explanation, including how it best fits the clinical picture and why it is more likely than the other options.</instruction>*

*<output_format>*

*<diagnosis_justification>*

*<fit_to_clinical_picture>[Explanation]</fit_to_clinical_picture>*

*<comparison_to_alternatives>[Explanation]</comparison_to_alternatives>*

*</diagnosis_justification>*

*</output_format>*

*</step>*

*<step>*

*<instruction>Counterfactual Thinking: For your top two diagnoses, explain how your reasoning would change if one key symptom were absent or significantly different.</instruction>*

*<output_format>*

*<counterfactual_analysis>*

*<diagnosis_1>*

*<name>[Diagnosis name]</name>*

*<changed_symptom>[Symptom changed]</changed_symptom>*

*<impact_on_reasoning>[Explanation of impact]</impact_on_reasoning>*

*</diagnosis_1>*

*<diagnosis_2>*

*<name>[Diagnosis name]</name>*

*<changed_symptom>[Symptom changed]</changed_symptom>*

*<impact_on_reasoning>[Explanation of impact]</impact_on_reasoning>*

*</diagnosis_2>*

*</counterfactual_analysis>*

*</output_format>*

*</step>*

*<step>*

*<instruction>Briefly explain why you ruled out each of the other options.</instruction>*

*<output_format>*

*<ruled_out_diagnoses>*

*<diagnosis>*

*<name>[Diagnosis name]</name>*

*<reason_for_ruling_out>[Explanation]</reason_for_ruling_out>*

*</diagnosis>*

*<!-- Repeat for other ruled out diagnoses -->*

*</ruled_out_diagnoses>*

*</output_format>*

*</step>*

*<step>*

*<instruction>Meta-cognitive Reflection: Reflect on your diagnostic process. What assumptions did you make? Are there any potential biases in your reasoning? How confident are you in your diagnosis and why?</instruction>*

*<output_format>*

*<meta_cognitive_reflection>*

*<assumptions>[List of assumptions made]</assumptions>*

*<potential_biases>[Discussion of potential biases]</potential_biases>*

*<confidence_level>[Statement of confidence]</confidence_level>*

*<confidence_reasoning>[Explanation of confidence level]</confidence_reasoning>*

*</meta_cognitive_reflection>*

*</output_format>*

*</step>*

*<step>*

*<instruction>If applicable, suggest what additional information or tests would be helpful to confirm the diagnosis or rule out others.</instruction>*

*<output_format>*

*<additional_information_needed>*

*<test>[Suggested test or information]</test>*

*<rationale>[Reason for suggesting this test/information]</rationale>*

*<!-- Repeat for other suggested tests/information -->*

*</additional_information_needed>*

*</output_format>*

*</step>*

*</case_analysis>*

*<final_instructions>*

*Remember to maintain a logical, step-by-step approach in your analysis and explanation. Your response should demonstrate clinical reasoning and diagnostic acumen. Ensure that your output follows the XML structure provided in each step.*

*</final_instructions>*

*</prompt>*

## System prompt – basic prompting

*Prompt 1: I’m writing a literature paper on the accuracy of CGPT of correctly identified a diagnosis from complex, WRITTEN, clinical cases. I will be presenting you a series of medical cases and then presenting you with a multiple choice of what the answer to the medical cases.*

*Prompt 2: Come up with a differential and provide rationale for why this differential makes sense and findings that would cause you to rule out the differential. Here are your multiple choice options to choose from and give me a detailed rationale explaining your answer.*

*[Insert multiple choices]*

*[Insert all Case info]*

*[Insert radiology description]*

# Supplementary results

## Sensitivity analysis with basic prompting

When using basic prompting, correct responses were provided for 95 (63%), 60 (40%), and 68 (45%) cases by llama-3.1-70b-versatile, llama-3.1-8b-instant, and mixtral-8x7b-32768, respectively. Llama-3.1-70b versatile was the only model that outperformed GPT-3.5 (Table S1).

There was a total of 150 question with 4 multiple choice options per question. When considering rates of TP, FP, TN, and FN, llama-3.1-70b-versatile outperformed GPT-3.5 whereas llama-3.1-8b-instant and mixtral-8x7b-32768 did not (p=0.0126, p=0.318, p=0.913, respectively; Table S1).

## Sensitivity analysis using advanced prompting in GPT-4.0 Turbo

When presenting GPT-4.0 Turbo with the advanced prompts, correct responses were provided for 100 (67%) cases and aligned with user provided answers in 104 (69%) cases. When comparing performance with advanced prompting for the LLMs chosen for this study, only llama-3.1-70b-versatile outperformed GPT-4.0 Turbo (p=0.0135), whereas llama-3.1-8b-instant (p=0.715) and mixtral-8x7b-32768 (p=0.399) did not. For 14 cases, GPT-4.0 Turbo did not provide an answer using advanced prompting.

When considering rates of TP, FP, TN, and FN, llama-3.1-70b-versatile outperformed GPT-4.0 Turbo whereas llama-3.1-8b-instant and mixtral-8x7b-32768 did not (p=0.0002, p=0.963, p=0.835, respectively; Table S2).

**Table S1.** Summary of LLM responses when using basic prompting techniques. P-values refer to Chi-Square tests performed between the LLMs selected by us and GPT-3.5 responses reported by Hadi et al. [11]

|  | llama-3.1-70b-versatile | llama-3.1-8b-instant | mixtral-8x7b-32768 | GPT-3.5^1^ |
| --- | --- | --- | --- | --- |
| Primary outcome assessment | | | | |
| Correct responses, n (%) | 95 (63) | 60 (40) | 63 (45) | 74 (49) |
| P-value | 0.0004 | 0.0145 | 0.1045 | Ref. |
| Response aligned with the response given by most Medscape users, n (%) | 90 (60) | 62 (41) | 72 (48) | 92 (61) |
| P-value | 0.81 | 0.000530 | 0.0204 | Ref. |
| Secondary outcome assessment | | | | |
| True positive, n (%) | 95 (16) | 60 (10) | 68 (11) | 73 (12) |
| False positive, n (%) | 55 (9) | 90 (15) | 82 (14) | 77 (13) |
| True negative, n (%) | 395 (66) | 360 (60) | 368 (61) | 373 (62) |
| False negative, n (%) | 55 (9) | 90 (15) | 82 (14) | 77 (13) |
| P-value | 0.0125 | 0.318 | 0.913 | Ref. |
| Accuracy, % | 82 | 70 | 73 | 74 |
| Precision, % | 63 | 40 | 45 | 49 |
| Sensitivity, % | 63 | 40 | 45 | 49 |
| Specificity, % | 88 | 80 | 82 | 83 |

LLM, large language models

^1^GPT-3.5 responses as reported by Hadi et al. [11]

**Table S2.** Summary of LLM responses. P-values refer to Chi-Square tests performed between the LLMs selected by us and GPT-4.0 Turbo responses using advanced prompting.

|  | llama-3.1-70b-versatile | llama-3.1-8b-instant | mixtral-8x7b-32768 | GPT-4.0 Turbo |
| --- | --- | --- | --- | --- |
| Primary outcome assessment | | | | |
| Correct responses, n (%) | 119 (79) | 97(65) | 93 (62) | 100 (67) |
| P-value | 0.0134 | 0.715 | 0.399 | Ref. |
| Response aligned with the response given by most Medscape users, n (%) | 94 (63) | 90 (60) | 97 (65) | 104 (69) |
| P-value | 0.222 | 0.0908 | 0.327 | Ref. |
| Secondary outcome assessment | | | | |
| True positive, n (%) | 119 (20) | 97 (16) | 93 (16) | 100 (17) |
| False positive, n (%) | 31 (5) | 53 (9) | 57 (10) | 50 (8) |
| True negative, n (%) | 419 (70) | 390 (65) | 390 (65) | 386 (64) |
| False negative, n (%) | 31 (5) | 60 (10) | 60 (10) | 64 (11) |
| P-value | 0.0002 | 0.963 | 0.835 | Ref. |
| Accuracy, % | 90 | 81 | 81 | 81 |
| Precision, % | 79 | 65 | 62 | 67 |
| Sensitivity, % | 79 | 62 | 61 | 61 |
| Specificity, % | 93 | 88 | 87 | 89 |

LLM, large language models
