# Supplementary material for: Evaluation of large language models as a diagnostic tool for medical learners and clinicians using advanced prompting techniques
Source: PLoS One. 2025 Aug 1;20(8):e0325803. doi: 10.1371/journal.pone.0325803 (PMC12316197; doi:10.1371/journal.pone.0325803)
Supplement: Attachment — Submitted filename: LLM - PLOS ONE - Response to reviewers 2.docx [file pone.0325803.s005.docx]

*The editor, and to a lesser extent the two reviewers, requested that you revise the paper. In my opinion, you have done this only rudimentarily. For example, you have not presented any alternative approaches as requested by the editor.*

*It is not clear to me why you did not follow Reviewer 2's recommendation to conduct the analysis with GPT-4. I would do this supplementary to GPT-3.5. This would make the comparison much more valuable.*

Response: We thank the reviewer for their feedback. In addition to the previously described sensitivity analysis using basic prompting for the three LLMs selected by us, we have provided results for a sensitivity analysis using advanced prompting strategies in GPT-4.0 Turbo (page 9 paragraph 1; supplementary materials, page 6, paragraph 3,4). Our findings demonstrate that advanced prompting strategies can lead to better results when applied to clinical practice scenarios even when smaller parameter models without specialized healthcare training or GPT-4.0 Turbo are used (page 13, paragraph 1).

*Here are a few further remarks:*

*Although you included the prompts in the appendix, the text does not describe the difference between basic and advanced promptings. It is also not clear from the text that the LMS were required to choose a diagnosis from four options.*

Response:

We thank the reviewer for their feedback and have provided additional context on the difference between advanced and basic prompting (page 12, paragraph 1). We have also clarified that LLMs were asked to select an answer from the Medscape multiple choice options (page 7, paragraph 1).

*The theoretical part of the paper is very short. This may be appropriate for the topic and the current state of research. But, I recommend adding more about the state of the research question under investigation. For example, in the introduction, you do not explain why GPT does not provide good answers and why other LMS should.*

Response:

We thank the reviewer for their suggestion. As stated in the introduction, the aim of our study was to demonstrate the role of LLMs as a diagnostic and educational tool using advanced prompt engineering. The focus was not to demonstrate inferior performance of ChatGPT, but rather show that using advanced prompt engineering, even smaller parameter LLMs can achieve accurate results. We chose open-source LLMs for this study for purposes of reproducibility. As discussed, our criticism on ChatGPT being used in previous studies is due to the fact that it is proprietary to OpenAI. Depreciation of previous ChatGPT models limits their access to future investigators and thus impacts reproducibility which is a cornerstone of research (page 13, paragraph 2). In fact, as shown in the sensitivity analyses, results using advanced prompting strategies were comparable between GPT-4.0 Turbo and the LLMs selected by us.

*The title states that the use of LLMs for diagnosis is intended for the training of medical learners and clinicians. This is not clearly stated in the article, but it is important for ethical and practical reasons.*

Response: As stated in the introduction, the aim of our study was to explore the role for LLMs as an educational tool (page 5, paragraph 1: “The aim of the current study is therefore to explore the potential role for LLMs as an educational tool for medical trainees by using three LLMs and advanced prompt engineering techniques.”).

*There is an error in the abstract (line 35): you report the 'correct response rate', but for GPT-3.5, the accuracy is reported (74% instead of 49%).*

Response: We have corrected this error in the abstract (page 2, paragraph 3).

*In line 93 of the methods section, you write "we demonstrate that even smaller to medium-sized models [...] can potentially outperform larger proprietary models". Shouldn't it say "we want to demonstrate"?*

Response: We have updated the text appropriately (page 6, paragraph 1).

*In line 163, you state that an "alpha of 0.5" is considered statistically significant. Shouldn't it say a p-value of 0.05 or 5%?*

Response: This error has been corrected (page 9, paragraph 1).

*In line 164, you state that they analyzed with R (4.2.1). Today, it is standard to provide a reference and also mention and cite the packages used. This is completely missing in your paper."*

Response: We have addended this information (page 9, paragraph 1).
